# The evolving art of creating genetic diversity: From directed evolution to synthetic biology

**DOI:** 10.1016/j.biotechadv.2021.107762

**Published:** 2021

**Authors:** Andrew Currin, Steven Parker, Christopher J. Robinson, Eriko Takano, Nigel S. Scrutton, Rainer Breitling

**Affiliations:** Manchester Centre for Synthetic Biology of Fine and Speciality Chemicals (SYNBIOCHEM), Manchester Institute of Biotechnology, The University of Manchester, Manchester M1 7DN, United Kingdom

**Keywords:** Mutagenesis, Pathway assembly, Genetic engineering, Directed evolution, Synthetic biology, Metabolic engineering, PCR, Polymerase Chain Reaction, LCR, Ligase Cycling Reaction, SDM, site-directed mutagenesis, OE-PCR, overlap extension PCR

## Abstract

The ability to engineer biological systems, whether to introduce novel functionality or improved performance, is a cornerstone of biotechnology and synthetic biology. Typically, this requires the generation of genetic diversity to explore variations in phenotype, a process that can be performed at many levels, from single molecule targets (*i.e.*, in directed evolution of enzymes) to whole organisms (*e.g.*, in chassis engineering). Recent advances in DNA synthesis technology and automation have enhanced our ability to create variant libraries with greater control and throughput. This review highlights the latest developments in approaches to create such a hierarchy of diversity from the enzyme level to entire pathways *in vitro*, with a focus on the creation of combinatorial libraries that are required to navigate a target's vast design space successfully to uncover significant improvements in function.

## 1 Introduction

Diversity is the foundation upon which the biological world is built, enabling Darwinian evolution through iterative cycles of genetic variation and selection. Through this process natural selection has produced countless biological macromolecules with different functions, including catalysis (*e.g.* enzymes), molecular recognition (*e.g.* antibodies), structural (*e.g.* cytoskeletal proteins) and signalling (*e.g.* hormones). This dramatic demonstration of the potential for harnessing genetic diversity to unearth specialised performance and novel functionality has inspired the powerful technique of directed evolution (DE), which is now commonly employed to engineer improvements in enzyme performance. Mimicking the process of natural evolution, DE performs iterative cycles of DNA mutagenesis and selection/screening on a specific genetic target in order to isolate new enzyme variants with improved function (Dougherty and Arnold, 2009; Tracewell and Arnold, 2009).

DE has become a vital tool in the development of biocatalysts for industrial processes, engineering improvements such as activity towards non-native substrates, increased turnover numbers, *k*_cat_, and improved solvent stability (Cheng et al., 2015; Cherry and Fidantsef, 2003; Dougherty and Arnold, 2009). But, clearly, natural selection operates on a wide hierarchy of different levels, from individual genes to entire organisms. Thus, whilst DE typically targets a single enzyme-coding sequence, its concepts should be equally applicable at higher levels of hierarchy, for example when optimising the overall function of an integrated biological system, such as a biosynthetic pathway within an heterologous host. Fortunately, our ability to engineer larger genetic constructs containing multiple components has advanced dramatically in the past decade through synthetic biology, bringing such an ambitious expansion of the scope of DE within reach of the experimentalist.

Synthetic biology can have diverse and creative applications, ranging from biosynthesis of natural and non-natural products, to biofuels and engineered organisms for healthcare (Breitling and Takano, 2015; Khalil and Collins, 2010; Wu et al., 2019a). Progress has been driven by the increasing throughput and decreasing cost of DNA synthesis, making synthetic DNA available to every laboratory. Coupled with widespread adoption of automation (Chao et al., 2017; Hillson et al., 2019) this allows for the assembly and engineering of whole metabolic pathways (Ellis et al., 2011), and has enabled the emergence of synthetic genomics, where organisms with entirely synthetically-derived genomes are constructed (Foo and Chang, 2018; Gibson et al., 2008; Gibson et al., 2010; Pretorius and Boeke, 2018). The high-throughput capabilities afforded by automation enable many diverse pathway and genomic sequences to be created, which can then be tested to identify an optimally performing chassis by utilising a design–build–test–learn framework (Carbonell et al., 2018). This is required because, as with traditional DE, the component parts may not function efficiently within their new chassis, and engineering these pathways could improve overall performance (Carbonell et al., 2016; Chi et al., 2019; Decoene et al., 2018). However, other additional factors may hinder performance, such as interference with host metabolism, supply of substrates *in vivo* and dynamic flux control (David et al., 2016; Gerosa and Sauer, 2011; Solomon et al., 2012; Wu et al., 2015). Consequently, optimisation of these chassis also requires genome editing and engineering (for instance, gene knock-outs and knock-ins, gene overexpression, and relaxation of feedback inhibition) to alter fluxes and improve overall performance (*e.g.*, rate, yield and titre of the target compound production). Genetic diversity can be achieved through genome engineering (employing techniques like CRISPR, recombineering, zinc-finger nucleases) *in vivo* and are often organism-specific. These approaches have recently been reviewed elsewhere (Naseri and Koffas, 2020; Simon et al., 2019), and are not discussed in this review. Here we focus on recent developments in the generation of genetic diversity at multiple levels *in vitro*, typically introducing designed alterations *via* synthetically-derived DNA, which are widely used in biotechnology and synthetic biology.

For any bio-engineering project the challenge facing the experimenter is the same: the total number of coding sequences and their combinations that could be created is many orders of magnitude larger than what it is possible to create and test in the laboratory. This concept is described as sequence space (Currin et al., 2015; Hayashi et al., 2006; Povolotskaya and Kondrashov, 2010; Wong et al., 2007), which describes the total number of possible variants of a given sequence of length *n*. Sequence space is vast and experimentally untestable for even very small protein sequences, for example the sequence space of a small 100 amino acid protein is ~1.3 × 10^130^ (20^100^, given the 20 possible amino acids), a number larger than the number of atoms in the observable universe (Kondrashov and Kondrashov, 2015). The combinatorial explosion further accelerates when multiple proteins functionally interact in a complex biological system, and a variety of regulatory elements add a further level of complexity to the challenge. Inevitably, this results in the need to reduce the breadth of the experiments to limit the search to a subset of the total diversity that can be effectively screened in the laboratory (Romero and Arnold, 2009; Romero et al., 2013). Unfortunately, despite the wealth of sequences, characterisation data and computational tools available, the behaviour of biological sequences remains largely unpredictable, with the exact nature of beneficial mutations still needing to be empirically determined, often through screening as many variants as possible. The experimenter must therefore select where and how to mutate their target sequence (or sequences), which can be assisted by prior knowledge, structural and protein characterisation data, and computational tools. This challenge is made dramatically more complicated by the phenomenon of epistasis, which describes the interdependence of amino acids throughout the sequence (even between different sequences within a biological system). Consequently, the beneficial effect of one mutation can be dependent on another mutation elsewhere (Miton and Tokuriki, 2016; Sailer and Harms, 2018; Storz, 2018). This is supported by the frequent observations that single-point mutations are rarely sufficient to elicit significant changes in performance (*e.g.* activity or productivity) (Reetz et al., 2006), and the control of pathway flux is typically shared widely among the enzymes in a reaction cascade. This review highlights the considerable recent progress in approaches for the creation of combinatorial diversity in DNA sequences. In particular, we discuss the developments in creating DNA diversity *in vitro* using specifically controlled (targeted rather than random) techniques. Whilst not specifically reviewed here, this area has significant overlap with the topics of DNA synthesis (oligonucleotide and gene synthesis) and assembly (cloning and synthetic genomics). In this review we elucidate that genetic diversity creation *in vitro* is a vital strategy to engineer biological macromolecules, with diverse applications in biotechnology and synthetic biology.

## 2 Creation of random genetic diversity

Early pioneering work on DE by Frances Arnold and co-workers utilised random mutagenesis methods to create diversity, prior to screening for improved enzyme function (Arnold, 1993; Chen and Arnold, 1991; Chen and Arnold, 1993). Due to its simplicity of use, by far the most popular random approach is error-prone polymerase chain reaction (epPCR), whereby mutations are introduced by a Taq polymerase during PCR amplification (McCullum et al., 2010; Wong et al., 2006). The method is generic (as no prior information on protein structure or function is needed) and optimised protocols are available that reduce mutation bias and provide tuneable control over mutation frequency (Biles and Connolly, 2004; Cadwell and Joyce, 1992; Copp et al., 2014) and general location (Yang et al., 2017). However, epPCR is limited in many regards: mutation of two consecutive bases is rare (meaning not all possible amino acids can be encoded at a given codon), it typically requires large screening efforts, stop codons can be encoded (3.2% of all possible single-base mutations within codons), and it cannot specifically mutate selected bases. Targeted mutagenesis methods overcome these limitations, providing highly accurate and controllable mutations of any desired sequence; these methods are the focus of this review.

Noteworthy alternative random diversity creation methods include recombination, exemplified by Stemmer's ‘DNA shuffling’ technique (where homologous sequences are fragmented and reassembled (Kikuchi et al., 1999; Stemmer, 1994a; Stemmer, 1994b; Zhao and Arnold, 1997)) and *in vitro* genome rearrangement using Synthetic Chromosome Rearrangement and Modification by LoxP-mediated Evolution (SCRaMbLE), mediated by a Cre recombinase and resulting in random recombination between loxP sites (Jia et al., 2018; Ma et al., 2019; Wu et al., 2018).

## 3 Targeted diversity creation

Variant sequence libraries encoding targeted (designed) mutations can be assembled through a number of different approaches *in vitro*, though universally these methods utilise synthetic variant oligonucleotides to determine the mutations through their incorporation into newly synthesised DNA. In the past decade, advances in oligonucleotide synthesis have dramatically changed the way that targeted mutations are created.

### 3.1 Variant library design and strategies for sequence diversification

Sequence libraries can be synthesised using a variety of methods to encode specifically designed variants. Given this control, attention must be given to the nature of mutations created and the number of variant sequences this creates. It is often desirable to create many mutations to test for a desired function, however simultaneous mutation at multiple sites within a protein can create a library of variant sequences which is impractically large for screening. For example, simultaneous mutation of just three amino acids (to any of the 20 amino acids) generates 8000 (20^3^) protein variants, requiring significant high-throughput screening to experimentally validate. Mutation of further residues in the same way grows the library size exponentially, with just seven randomised residues creating a library size of 1.28 × 10^9^ (20^7^) variants, beyond even ultra-high throughput screening capabilities (Agresti et al., 2010; Autour and Ryckelynck, 2017). Careful design of combinatorial variant libraries is therefore crucial to rationally reduce the experimental screening burden (Qu et al., 2020) and powerful computational tools are now available for the selection of amino acids for mutation using phylogenetic and molecular simulation approaches (Goldenzweig et al., 2016; Khersonsky et al., 2018; Sumbalova et al., 2018; Weinstein et al., 2020).

As outlined in section 3.2, oligonucleotides from solid-phase synthesis can be designed to remove amino acid bias (over-representation of certain amino acids) in variant libraries. In addition, a number of approaches are also available to reduce codon redundancy (Pines et al., 2015). For example, the ‘22c-trick’ (Kille et al., 2013) reduces redundancy and bias by using three variant codons (NDT, VHG, TGG), incorporated into three separate oligonucleotides, together encoding all 20 amino acids with 22 codons and no stop codons (an improvement compared to 32 codons for NNK randomisation). However, one limitation for this approach is that there can be no overlap between mutagenic oligonucleotides for each target codon, as randomisation of two codons using the same primer sequence would require synthesis of 3^2^ = 9 different oligonucleotides. Mutagenesis of consecutive residues without redundancy or bias has been achieved using the Sloning method (Van den Brulle et al., 2008) and its successor ProxiMAX (Ashraf et al., 2013), employing an alternative ligation and restriction digest-based approach to library synthesis.

Various *in silico* tools have been created to reduce redundancy (or codon compression) and also to assist in the design of bespoke degenerate codons that encode only those amino acids required, including CodonGenie (Swainston et al., 2017), ANT (Engqvist and Nielsen, 2015), SwiftLib (Jacobs et al., 2015), DYNAMCC (Halweg-Edwards et al., 2016) and DC- or MDC-Analyzer (Tang et al., 2012; Wang et al., 2015). A generic ‘smart’ reduced codon (NDT, whereby N = A, T, G or C; D = A, G or T) was developed by Reetz et al. (Qu et al., 2020; Reetz et al., 2008), encoding 12 possible codons and amino acids (F, L, I, V, S, T, H, N, D, C, R, G) covering a range of physicochemical properties. These approaches seek to reduce library size through the rational design of selected variant sequences, easing the burden of screening duplicate or unwanted variants. The notion of ‘smart’ libraries has also been addressed by the GeneORator method (Currin et al., 2019), an approach based on Boolean logic that mutates multiple codons in different combinations (so called ‘OR-type’ mutations), in order to create rationally reduced combinatorial libraries. (See Table 1.)

**Table 1:** A summary of the library design approaches outlined in this review.

Category	Method	Features	Reference
Protein variant design	PROSS, FuncLib, HotSpot Wizard	Automated algorithms for the design of protein variants.	(Goldenzweig et al., 2016; Khersonsky et al., 2018; Weinstein et al., 2020)
Variant codon design	CodonGenie, ANT, SwiftLib, DYNAMCC, DC- and MDC-Analyzer	*In silico* tools for degenerate codon design and analysis.	(Engqvist and Nielsen, 2015; Halweg-Edwards et al., 2016; Jacobs et al., 2015; Swainston et al., 2017; Tang et al., 2012; Wang et al., 2015)
	22c-trick	Reduces redundancy bias by using three variant codons (NDT, VHG, TGG) for randomisation.	(Kille et al., 2013)
	NDT codon	Generic ‘smart’ reduced codon encoding amino acids F, L, I, V, S, T, H, N, D, C, R and G.	(Qu et al., 2020; Reetz et al., 2008)
Library design	GeneORator	Use of Boolean logic to create large combinatorial libraries with rationally reduced complexity.	(Currin et al., 2019)
RBS library design	RedLibs	A “reduced libraries” approach for the design of RBS libraries for pathway engineering.	(Jeschek et al., 2016)
Cloning design	Potapov et al.	Golden gate four base pair overhang design tool.	(Potapov et al., 2018)

### 3.2 DNA oligonucleotide synthesis

**Fig. 1:**
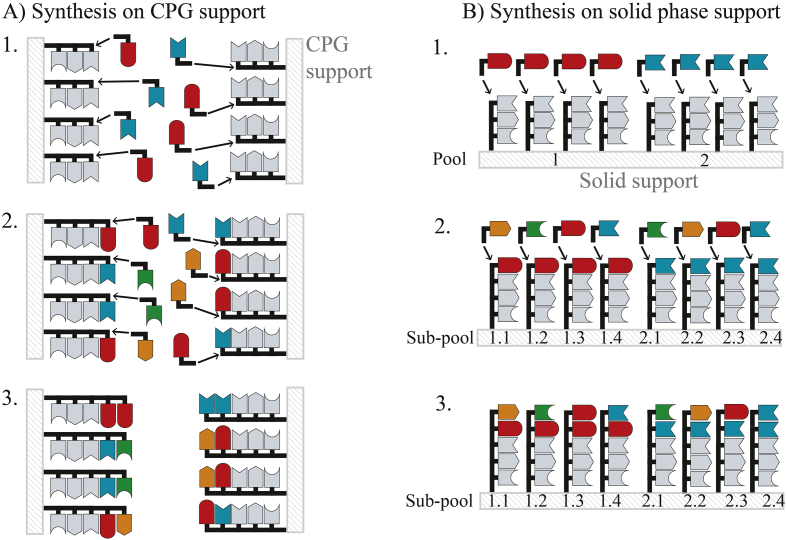
Two approaches for the synthesis of DNA oligonucleotide libraries. For each method, grey denotes non-variant (non-mutagenic) nucleotides and colour represents variant (mutagenic) nucleotides that create an oligonucleotide library. A) Conventional synthesis using controlled pore glass (CPG) adds a mixture of nucleotides for polymerisation at a variant position (*e.g.* red and blue in 1. and red, green, blue and orange in 2.) to create an oligonucleotide pool with all possible nucleotide combinations (3.). B) Synthesis of oligonucleotides using solid phase support, where specific single nucleotides are added to each pool (red to pool 1 and blue to pool 2 in 1.) and sub-pool (each orange, green, red and blue to separate sub-pools in 2.) to individually synthesise each desired variant sequence (3.). (For interpretation of the references to colour in this figure legend, the reader is referred to the web version of this article.)

In the past decade advances in DNA oligonucleotide synthesis technology have altered how genetic diversity can be created. Oligonucleotides are short (typically <100 nt in length) sequences of single stranded DNA, synthetically derived using phosphoramidite chemistry (Caruthers et al., 1987; Itakura et al., 1984). Oligonucleotides are employed both as primers for PCR and as overlapping building blocks for assembly of larger synthetic DNA sequences by gene synthesis (Czar et al., 2009; Hughes et al., 2011; Ma et al., 2012), and can be designed to be the source of controlled variant sequences for diversity creation. Established methods for oligonucleotide synthesis utilise controlled pore glass (CPG) supports from which the synthesised strand is elongated (Fig. 1A). Variant library oligonucleotides are created by incubating mixtures of phosphoramidite nucleosides (*e.g.* an equal mixture of all four nucleosides) for each chosen variant position.

Technological advances to miniaturise phosphoramidite chemistry using solid-phase supported synthesis (LeProust et al., 2001; LeProust et al., 2010; Tian et al., 2004) utilise inkjet ‘printing’ techniques to deliver reagents for polymerisation on a miniscule scale. This technology reduces synthesis volumes by >10^6^ (to sub-femtomole scale (Li et al., 2019)) and thus permits thousands of individual oligonucleotides sequences to be synthesised in parallel at low cost. Consequently, each individual variant sequence of a library can be specifically synthesised in parallel, which when cleaved from the solid support creates a pool of oligonucleotides containing only the desired variant sequences (Fig. 1B). This provides tuneable control over the variant nucleotides at each position within the library and their relative frequency, which has been exploited to reduce codon redundancy (use multiple codons for the same amino acid) and bias (Acevedo-Rocha et al., 2015; Cleary et al., 2004; Hoebenreich et al., 2014; Li et al., 2018; Sayous et al., 2020). For example, randomisation of a codon using a single oligonucleotide is typically done by choosing the NNK codon (N = A, T, G or C; K = G or T), which encodes all 20 amino acids using 32 codons, but with unnecessary redundancy for 8 amino acids (L, V, P, A, G, S, T and R) and a stop codon (Nov, 2012). Furthermore, amino acid bias exists for the NNK codon, with three codons each for R, L and S, and just one codon each for twelve other amino acids. This bias becomes more pronounced as the number of randomised codons increases, leading to dramatic under-representation of some variants in the library (consequently increasing the experimental screening burden and the likelihood that beneficial mutations will be missed). Solid-phase synthesis can synthesise just the desired 20 codons for full randomisation, and several studies have demonstrated the value of reducing bias to improve library quality for directed evolution (Li et al., 2018; Sayous et al., 2020).

Whilst solid-phase oligonucleotide synthesis offers several advantages, some limitations are notable. First, the sub-femtomole synthesis scale generates only limited material compared to the larger CPG scales, although amplification of these samples has been reported (Schmidt et al., 2015) this remains a limitation for some applications. Second, the costs for oligo library pools are substantially higher (roughly 10-fold) than those for pools generated using CPG synthesis (these are currently only available through Twist Bioscience).

### 3.3 Creating sequence diversity for directed evolution and synthetic biology

Since its inception by Smith and colleagues in 1978 (Hutchison et al., 1978), site-directed mutagenesis (SDM) has become an indispensable tool in biotechnology. The most commonly used mutagenesis methods utilise the polymerase chain reaction (PCR), amplifying template sequences with mutagenic oligonucleotide primers. Conventional methods of SDM, such as QuikChange (Mao et al., 2011; Xia et al., 2015), amplify the entire target sequence (and plasmid backbone) to introduce mutations in a single PCR step. This approach has variable efficiency and yields and, despite improvements to improve robustness (notably the commercial Q5 and Phusion mutagenesis kits), is limited by its inability to create mutations at more than one contiguous region. These approaches are therefore being superseded by more robust and efficient methods capable of creating combinatorial libraries.

As outlined above, approaches to creating genetic diversity have historically been focussed on the synthesis of variant libraries of gene sequences for directed evolution. However, the emergence of synthetic biology has expanded the challenge of engineering biology to a greater scale, to include whole biosynthetic gene pathways (Smanski et al., 2014; Smanski et al., 2016) and genomes (Eisenstein, 2020; Schindler et al., 2018). The genetic components handled in synthetic biology are preferably standardised as ‘parts’ and can include elements with regulatory functions (*e.g.* promoters, ribosome binding sites and terminators) as well as coding sequencing (*e.g.* genes). For DNA assembly *in vitro*, multi-part constructs several kilobases in size are often required, in order to create novel biosynthetic pathways to function within a desired host organism (chassis) (Casini et al., 2015; Ellis et al., 2011). Unfortunately, the *a priori* design of an optimal metabolic pathway for a particular chassis is currently an unrealistic aim as, alongside the challenges described for traditional DE, there are additional layers of complexity that can require optimisation. For novel pathways encoding multiple heterologous genes these include gene order, transcription and translation control, and construct copy number. Furthermore, imbalance in metabolic fluxes can cause growth inhibition, increased metabolic burden and production of toxic intermediates, leading to suboptimal yields of target compounds (Dueber et al., 2009; Jeschek et al., 2017; Wu et al., 2016b). Consequently, iterative variations on pathway designs should be tested to determine optimised constructs, with efforts focussed on the ability to quickly and efficiently create combinatorial variations of assembled parts.

A number of experimental approaches have been established to conduct the multifactorial optimisation of metabolic pathways for synthetic biology, including multivariate modular metabolic engineering (MMME (Biggs et al., 2014)) and the creation of small high-content libraries (*e.g.* using promoter libraries or ribosome binding site engineering), as reviewed recently (Jeschek et al., 2017). For example, the RedLibs algorithm was developed to design RBS libraries for pathway optimisation (Jeschek et al., 2016). Particularly noteworthy is the Design of Experiments (DoE) strategy, a generic approach to statistically assess the effect of individual factors in affecting overall performance in order to avoid more exhaustive sampling of sequence space. In a synthetic biology setting this has been successfully applied to optimise sequence and assembly order of coding and regulatory parts as well as general experimental conditions (Carbonell et al., 2018; Xu et al., 2017; Zhou et al., 2015). DoE therefore reduces the exploration space down to a number of assemblies that can realistically be assembled and screened in a high throughput laboratory. In this review we discuss the experimental methodologies that can be employed to conduct these combinatorial optimisation experiments.

Many methods are available for the assembly of large DNA constructs from modular parts. Given that multiple parts are often assembled simultaneously, order and directionality must be controlled, which is typically done using either overlapping homologous sequences (*e.g.* MODAL (Casini et al., 2013), BASIC (Storch et al., 2015), PaperClip (Trubitsyna et al., 2014; Trubitsyna et al., 2017), USER (Genee et al., 2015; Geu-Flores et al., 2007; Nour-Eldin et al., 2006), Gibson (Gibson et al., 2008; Torella et al., 2014), DNA assembler (Shao and Zhao, 2013; Shao et al., 2009; Yuan et al., 2016), TEDA (Xia et al., 2019) and DATEL (Jin et al., 2016a)), or single strand overhangs created by restriction endonuclease digestion (*e.g.* Golden Gate (Engler et al., 2008; Gao et al., 2013)). Whilst many of these methods are capable of assembling large DNA constructs with many parts, not all are efficient and flexible enough for creating diverse combinatorial variant pathways.

#### 3.3.1 Restriction enzyme-based methods

**Fig. 2:**
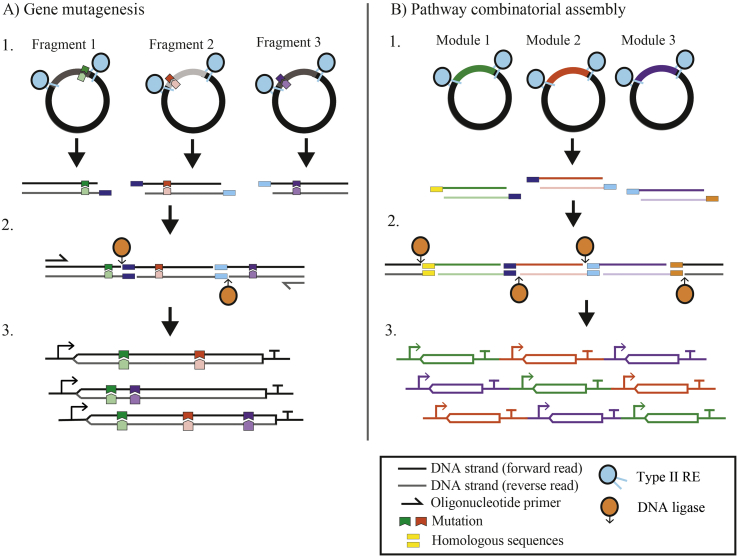
Sequence diversification using restriction enzyme-based methods. Techniques to create libraries for both A) single genes and B) pathways follow the same general procedure. 1. Restriction digestion of the target sequence using a type IIS restriction endonuclease (*e.g. Bsa*I). 2. Ligation is performed using a DNA ligase with directionality controlled by the single-stranded overhang sequences to yield (3.) gene and pathway combinatorial libraries.

For DE, routine cloning-based strategies have also been employed, using restriction enzymes followed by ligation to assemble different DNA fragments. These approaches provide a means to “mix and match” different gene fragments to create diversity (Fig. 2A). Encoding variants within these fragments provides a rapid means to test different combinatorial mutations; however, the limited ligation efficiency limits these approaches to relatively small (<10^4^) libraries (Popova et al., 2015; Quaglia et al., 2017). (see Table 2, Table 3.)

**Table 2:** A summary of the mutagenesis methodologies outlined in this review. Efficacy is defined as the proportion of transformant cells encoding any mutations. Efficiency is defined as the proportion of the mutated transformant cells with *all* desired sequences or mutations. NR highlights where data is not reported in the original publication.

Category	Method	Features	Efficacy	Efficiency	Reference
Restriction enzyme-based	Type IIS restriction enzyme (BsaI) modular assembly	Mix-and-match assembly of variant gene fragments *via Bsa*I digestion and DNA ligation, similar to Golden Gate cloning. Limited to small library sizes.	100%	n.a.	(Popova et al., 2015; Quaglia et al., 2017)
	VersaTile	Mix-and-match assembly of protein modules, based on Golden Gate assembly.	95%	n.a.	(Gerstmans et al., 2020)
Ligation-based	Darwin assembly	Mutations encoded by oligonucleotides are incorporated by DNA polymerase and ligase activities. Method has high efficiency for multiple mutation sites.	98–100%	100%	(Cozens and Pinheiro, 2018)
	ProxiMax	Ligation of defined codons to build a library without codon bias. Requires trimer oligonucleotides, robust and controllable but becomes challenging for multiple mutation sites.	NR	100%	(Ashraf et al., 2013)
PCR-based	QuikChange (and variations thereof)	Mutations introduced by a primer pair during inverse PCR. Limited to mutation of one position.	84%	55%	(Mao et al., 2011; Xia et al., 2015)
	Overlap extension PCR (OE-PCR)	A gene fragment is amplified by PCR (with primers adding both mutations and homologous termini), then the full-length sequence is assembled by overlap extension PCR. Method is robust and reliable, though cumbersome for multiple mutation sites.	>90%	100%	(An et al., 2005; Bryksin and Matsumura, 2010; Cheng et al., 2017; Heckman and Pease, 2007; Hussain and Chong, 2016; Wäneskog and Bjerling, 2014; Wei et al., 2012; Williams et al., 2014; Xiao and Pei, 2011)
	Asymmetric PCR	A single-stranded gene fragment is amplified by asymmetric PCR using mutagenic primers, which is then used as a megaprimer to introduce mutations into full sequence. Method is robust and reliable, though cumbersome for multiple mutation sites.	91–100%	100%	(Bi et al., 2012; Sadler et al., 2018)
	SpeedyGenes	Gene synthesis method, encoding mutations using the overlapping oligonucleotide primers that assemble the gene library *de novo*. Many combinatorial mutations can be efficiently assembled, though efficiency drops for large genes.	76–90%	100%	(Currin et al., 2017, Currin et al., 2014)

**Table 3:** A summary of the combinatorial pathway assembly methodologies outlined in this review. Efficacy is defined as the proportion of the transformant cells encoding any mutations. Efficiency is defined as the proportion of the mutated transformant cells with all desired sequences or mutations. Fidelity/accuracy is defined as the overall proportion of the transformant cells encoding the desired sequences or mutations. NR highlights where data is not reported in the original publication. Where possible, comparison data is reported for larger, multipart (>4) assemblies.

Category	Method	Features	Efficacy	Efficiency	Fidelity/ accuracy	Reference
Homologous overlap assembly	Gibson assembly	Assembly of fragments with >15 bp homologous overlaps in one-pot isothermal conditions with a T5 exonuclease, DNA polymerase and DNA ligase. Reliable assemblies though efficiency drops as more parts are assembled. Requires creation of 15 bp overlapping termini which increases sample preparation for combinatorial assemblies.	95%	87%	80–95%	(Gibson, 2011, Gibson, 2009; Gibson et al., 2009, Gibson et al., 2008; Torella et al., 2014)
	Modular Overlap-Directed Assembly with Linkers (MODAL)	Similar to Gibson assembly, extended to include modular reusable parts for combinatorial assemblies.	NR	NR	75–100%	(Casini et al., 2013)
	PaperClip	Unmodified parts are ‘clipped’ by ligation of specific double stranded oligonucleotides that confer the homologous sequences required to direct subsequent pathway assembly. Not scarless but more flexible for combinatorial assemblies.	NR	80%	NR	(Trubitsyna et al., 2017, Trubitsyna et al., 2014)
	Uracil Excision Cloning (USER)	Parts amplified using primers (containing uracil) are then cleaved using uracil DNA glycosylase to create overhangs, which then anneal for construct assembly. Increased sample preparation, like for Gibson method.	NR	94%	70–95%	(Cavaleiro et al., 2015; Genee et al., 2015; Geu-Flores et al., 2007; Nour-Eldin et al., 2006, p.)
	DNA assembler	Assembly of large multipart pathways *in vivo* using the homologous recombination capacity of *Saccharomyces cerevisiae*. Long homologous overlaps of parts are required for efficient assembly, increasing preparation and costs.	NR	NR	71 (44 kb assembly from 50 fragments) – 100% (12 kb assembly from 6 fragments)	(Shao et al., 2009; Shao and Zhao, 2013; Yuan et al., 2016)
	T5 Exonuclease-Dependent Assembly (TEDA)	Homologous overhangs are created by T5 exonuclease (as in Gibson assembly) allowing self-annealing, then repair and ligation occurs upon transformation into *E. coli*. Less reagents required compared to Gibson method, with similar efficiency.	NR	NR	>90%	(Xia et al., 2019)
	DNA Assembly with Thermostable Exonuclease and Ligase (DATEL and sDATEL)	Prepared parts encode 30 bp homologous termini, following denaturation and annealing Taq polymerase removes displaced overhangs and Taq ligase joins the juxtaposed termini. Up to 10 parts can be assembled, though with sample preparation limitations like other homology-based methods.	NR	NR	75%	(Ding et al., 2017; Jin et al., 2016a)
Restriction enzyme-based	Golden Gate assembly (also Scarless stitching)	One-pot assembly of multiple parts using BsaI digestion and DNA ligation. Directionality is controlled by the variable single-strand overhangs. Scarless and reliable but requiring increased sample preparation to repeatedly add different restriction sites/overhangs to parts for combinatorial assembly.	NR	NR	95–100%	(Engler et al., 2008; Gao et al., 2013; HamediRad et al., 2019; Smanski et al., 2014)
	MoClo, CIDAR MoClo, EcoFlex	Extension of Golden Gate method using multiple type IIS endonucleases, utilised for a hierarchical and modular assembly protocol. EcoFlex extends MoClo to encode pathway variants using multiple pre-assembly levels. Requires more time to prepare preassembled modules prior to assembly.	NR	NR	95–100%	(Iverson et al., 2016; Moore et al., 2016; Vecchione and Fritz, 2019; Weber et al., 2011; Werner et al., 2012)
	Biopart Assembly Standard for Idempotent Cloning (BASIC)	Utilises type IIS (*Bsa*I) restriction digestion of parts and then ligation to short oligonucleotide linkers to confer directionality of assembly, enabling more flexible and modular assemblies. Improved flexibility and sample preparation for combinatorial assemblies.	NR	90%	90%	(Storch et al., 2017, Storch et al., 2015)
	MIDAS	Utilises three type IIS endonucleases in three steps: 1) *Bsm*BI digestion and ligation forms source vectors, 2) BsaI digestion and ligation forms shuttle vectors, and 3) AarI and BsmBI digestion and ligation assembles the multigene constructs. Requires more time to prepare preassembled modules prior to assembly.	96%	90%	NR	(van Dolleweerd et al., 2018)
	Start-Stop assembly	Scarless assembly utilising the 3 bp start and stop codons to create expression units, followed by combinatorial assembly using BsaI digestion and ligation. Multistep protocol is longer compared to “one-pot” methods.	99%	NR	NR	(Taylor et al., 2019)
	Coussement et al.	Golden Gate method extended to include assembly of variant sequences using single strand assembly. Features similar to Golden Gate.	NR	NR	NR	(Coussement et al., 2017)
Ligation-based methods	RECODE	Similar to Darwin assembly for pathway libraries. Mutagenic oligonucleotides are incorporated into a new ssDNA strand (using a thermostable DNA ligase and polymerase during thermocycling), then PCR with terminal anchor primers create the dsDNA library. Efficiently creates combinatorial mutations independent of template pathway size, though does not rearrange part orders.	58%	100%	58%	(Jin et al., 2016b)
	Ligase Cycling Reaction (LCR)	Scarless assembly utilising a thermostable DNA ligase and bridging oligonucleotides, which direct assembly during thermocycling. Parts are modular as they do not require addition of homologous or restriction sites, permitting flexible combinatorial assemblies. Efficiency drops for larger multipart assemblies.	100%	80–100%	NR	(de Kok et al., 2014; Wiedmann et al., 1994; Yuan et al., 2016)

To date, the most popular approach to combinatorial pathway assembly utilises type IIS restriction endonucleases (*e.g. Bsa*I, *Bbs*I). These enzymes digest DNA to create single-stranded ‘sticky ends’ outside of their restriction site, permitting customisable overhangs (which confer order and directionality during the assembly of multiple parts) that can then be joined using T4 DNA ligase, as exemplified by the Golden Gate method (Fig. 2B). This requires the preparation of parts flanked by customised restriction sequences, which can be either encoded by the plasmid or added by PCR. Given that endonuclease digestion creates overhangs outside of the recognition sequence, ligated constructs are ‘scarless’ (devoid of any cloning-specific sequences). Many elaborations on Golden Gate have been developed, improving robustness and flexibility, including Goldenbraid and Scarless stitching (Smanski et al., 2014).

Several studies have demonstrated highly parallel assembly of combinatorial constructs using the Golden Gate approach, often through the standardisation of parts and linker sequences, notably MoClo (Vecchione and Fritz, 2019; Weber et al., 2011; Werner et al., 2012), CIDAR MoClo (Iverson et al., 2016), MIDAS (van Dolleweerd et al., 2018) and Start-Stop assembly (Taylor et al., 2019). Various studies have shown that optimised design of the single-stranded linkers is crucial for the efficient one-pot assembly of multiple parts when attempting Golden Gate assemblies (HamediRad et al., 2019; Potapov et al., 2018). Interestingly, Coussement *et al.* (Coussement et al., 2017) extended the approach to include single-strand assembly of variant sequences, creating promoter libraries that were then assembled into genetic pathways by Golden Gate to screen for increased pathway yield. Promoter and RBS libraries have also been assembled for screening using the EcoFlex methodology, a Golden Gate approach that uses multiple pre-assembly levels to build pathway variants (Moore et al., 2016). Recently, VersaTile utilised the Golden Gate approach to create random “mix-and-match” protein modules libraries (Gerstmans et al., 2020).

One limitation to Golden Gate approaches is the necessity to encode specific overhang sequences at each terminus to direct the order of the assembled parts. For combinatorial assemblies, where it is desirable to rearrange part order and have variable numbers of parts, unique overhang sequences must be generated for each part in each combination, which dramatically increases the workload for preparation of parts prior to assembly. This limitation is shared by other methods that depend on homologous sequences to confer directionality during DNA assembly, notably the isothermal assembly methods developed by Gibson et al. (Gibson, 2009; Gibson, 2011; Gibson et al., 2009). These methods are popular for the assembly of multiple DNA fragments and there are some examples of their use for combinatorial assemblies (Halleran et al., 2018; Santos-Moreno and Schaerli, 2019; Torella et al., 2014).

#### 3.3.2 Ligation-based methods

**Fig. 3:**
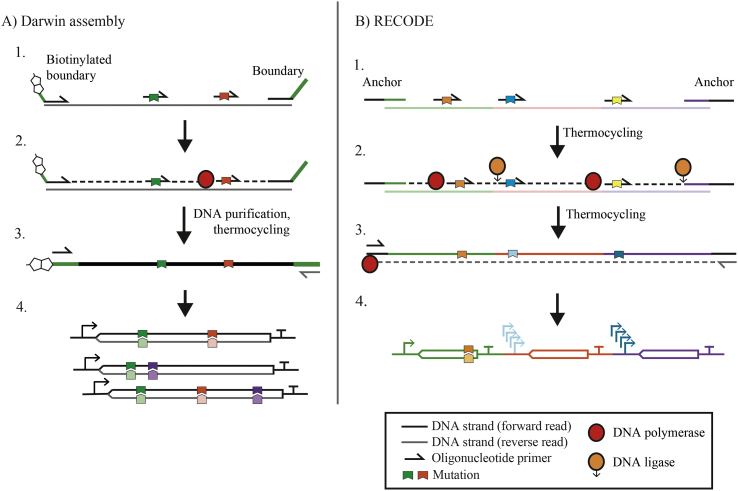
Combinatorial mutagenesis using polymerase extension and ligation. Both A) Darwin assembly and B) RECODE follow a similar protocol. 1. Oligonucleotide primers anneal to the target sequence, together with boundary/anchor oligonucleotides at each terminus. 2. DNA polymerase then polymerises from this primer to create the remaining non-mutated sequence up to the next anneal oligonucleotide, whereby the juxtaposed termini are ligated. 3. Polymerase amplification of the new mutated strands produces the (4.) desired variant library.

DNA concatenation *via* ligation is an efficient approach for mutation of multiple sites. Rather than using mutagenic oligonucleotides as PCR primers, they are instead hybridised to a template sequence, a polymerase then extends the oligonucleotide to fill in the gaps between hybridised oligonucleotides to create adjacent termini that are joined together by a DNA ligase. This approach provides a more attractive “one-pot” approach compared to PCR-based methods, which is advantageous when looking to increase throughput using automation. The Darwin assembly method (Cozens and Pinheiro, 2018) recently combines the ligation-based method with streptavidin-biotin purification to create a streamlined workflow, with an efficiency suitable to create large (10^8^ variants) and diverse combinatorial libraries (mutating up to 10 separate sites simultaneously, Fig. 3A).

The majority of synthetic biology approaches for the assembly of diverse pathway sequences focus on the modular assembly of multiple parts in different orders, in contrast to DE approaches to create gene libraries of variant sequence. However, Jin et al. (Jin et al., 2016b) described a ligation and PCR-based approach that is compatible for both gene and pathway variant libraries, called ‘rapidly efficient combinatorial oligonucleotides for directed evolution’ (RECODE, Fig. 3B). Through hybridisation of multiple mutagenic oligonucleotides that can bind anywhere within a pathway construct, combinatorial libraries were created whilst simultaneously mutating RBS, promoter and gene sequences for screening of compound yield. RECODE could be considered as a complementary approach to modular assembly methods like Golden Gate as, whilst it is unable to alter part orders, it possesses advantages in creating larger combinatorial variant libraries by mutating multiple sites across a pathway simultaneously.

**Fig. 4:**
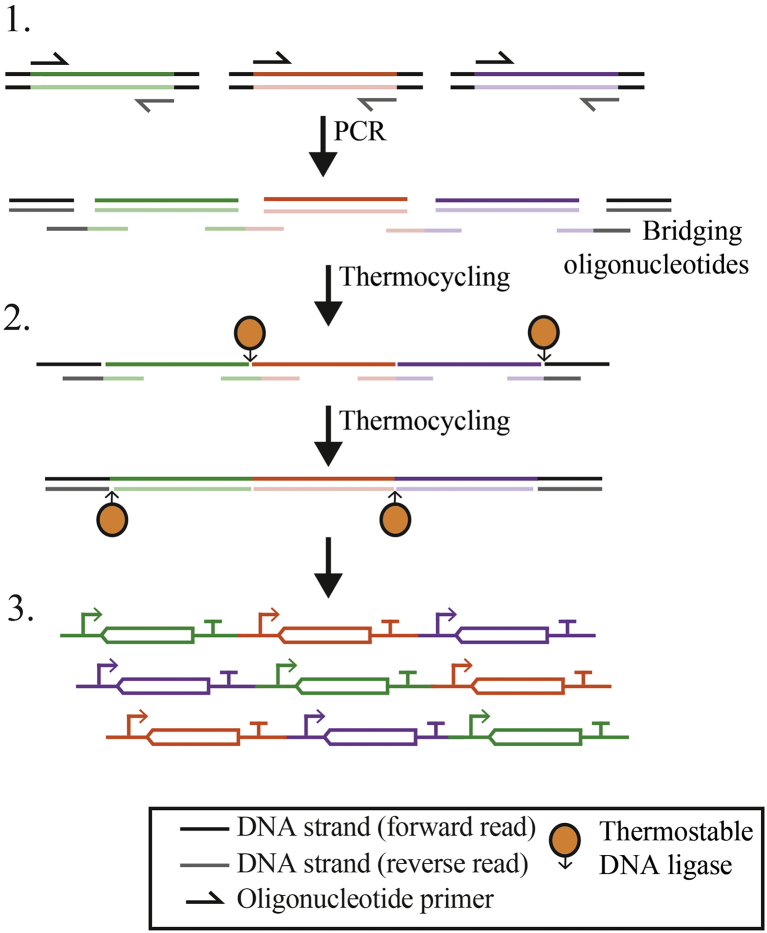
Construction of combinatorial pathway assemblies using ligation. LCR provides flexible assembly of pathways without homologous overhang sequences. 1. Target part sequences are amplified using PCR. 2. Parts are combined with a thermostable DNA ligase and “bridging” oligonucleotides, which provide the directionality to control the order of the assembled parts. Thermocycling allows bridging oligonucleotides to anneal to their target sequence, allowing juxtaposed DNA termini to be joined using by the ligase. Repeated cycling leads to the ligation of both strands to generate the desired pathway sequence.

For combinatorial pathway assemblies, the Ligase Cycling Reaction (LCR) approach does not require any addition of terminal sequences to each part (unlike restriction enzyme-based methods like Golden Gate). The directionality of LCR is controlled using bridging oligonucleotides, which are complementary to the terminal 5′ and 3′ sequences of the two parts to be joined (Fig. 4). Through repeated thermocycling, these oligonucleotides hybridise two target strands, creating adjacent 5′ and 3′ termini that are then ligated using a thermostable ligase (de Kok et al., 2014; Wiedmann et al., 1994). This approach means that the preparation of parts is the same for all assemblies and therefore each part only needs to be prepared once, providing a flexible platform on which to create countless combinatorial assemblies (Robinson et al., 2018). Automation of this approach has enabled high-throughput DoE studies as a means to optimise the biosynthesis of a number of natural products and commodity chemicals in *E. coli* (Carbonell et al., 2018; Robinson et al., 2020). Whilst LCR provides the most flexible assembly platform, its efficiency reduces for larger assemblies (>12 kb plasmids), a difficulty that Yuan et al. (Yuan et al., 2016) overcame by combining LCR with the ‘DNA assembler’ method in *Saccharomyces cerevisiae*, which assembled plasmids of 44 kb with an impressive 71% efficiency.

#### 3.3.3 PCR-based methods

**Fig. 5:**
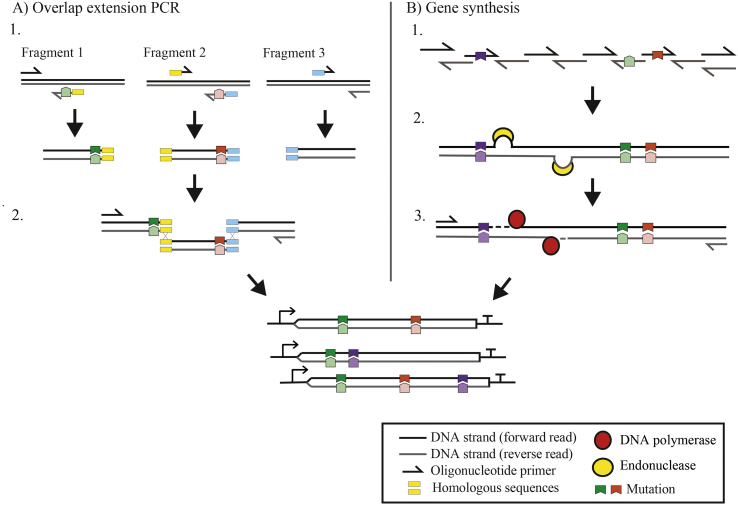
Synthesis of combinatorial libraries using PCR-based methods. A) OE-PCR (1.) first amplifies the gene fragments using oligonucleotide primers that encode both mutations and homologous terminal sequences. 2. Joining of the fragments is controlled by homologous sequences priming overlap extension during the second PCR. B) Gene synthesis assembles the desired sequence by (1.) OE-PCR, encoding mutations on the overlapping oligonucleotide primers. 2. Following an error correction step using an endonuclease the (3.) full length library sequence is assembled in a second PCR step.

Mutagenesis using overlap extension PCR (OE-PCR) is a popular two-step process for DE, whereby truncated gene amplicons are created using mutagenic PCR primers, which are then assembled together in a second step to create the full target sequence library (Bryksin and Matsumura, 2010; Heckman and Pease, 2007; Hussain and Chong, 2016; Williams et al., 2014; Xiao and Pei, 2011) (Fig. 5A). Further developments in this approach have made it amenable to combinatorial mutations (An et al., 2005; Cheng et al., 2017; Wäneskog and Bjerling, 2014; Wei et al., 2012). Similarly, mutagenesis using asymmetric PCR employs two steps, where mutagenic primers are used to create single-stranded products, which are then deployed as megaprimers to introduce these mutations in the second step (Bi et al., 2012; Sadler et al., 2018). Interestingly, pathway assembly by OE-PCR is not widely used. Trial experiments with OE-PCR in our laboratory have shown that the necessary long-range PCR runs have variable efficiency and this may account for the method's apparent unpopularity in large pathway assemblies (unpublished results).

Gene synthesis, whereby gene sequences are assembled from short oligonucleotides *de novo* (without a template), can also be as achieved using OE-PCR (Fig. 5B). Here, each oligonucleotide overlaps with the next, such that these short fragments can be stitched together during PCR to create an entire gene. As the gene is assembled *de novo* each time, mutations can be encoded by any oligonucleotide sequence in any position (providing they do not fall where oligonucleotides overlap), permitting large and diverse combinatorial libraries to be synthesised (Currin et al., 2014; Currin et al., 2017). This is an efficient process for DNA assemblies of less than 2 kb; however, due to the inherent error rate (arising from the oligonucleotide synthesis chemistry) the efficiency of larger assemblies drops significantly. Consequently, gene synthesis is typically used as the means to generate the synthetic biology ‘parts’, which are then assembled into larger constructs using the alternative methods outlined above.

## 4 Discussion

This review highlights recent developments in the creation of genetic diversity *in vitro*, ranging from large libraries of single gene targets for traditional DE to the assembly of larger combinatorial pathway libraries for systems-level DE by synthetic biology. For all approaches, the challenge of creating sufficient diversity to capture the desired functional improvement is a difficult one, given the potential sequence space and inevitable limitations on experimental throughput. Various methodologies have been outlined that attempt to address this issue, including reducing the necessary experimental effort (*i.e.* reducing bias and redundancy of gene libraries), creating ‘smart’ libraries of reduced diversity (restricting the search space to statistically designed variants), as well as improving process efficiency through new methods and automation.

A common issue for synthesis of genetic diversity *in vitro* is the transfer of this material to a screening platform *in vivo*, often achieved through transformation of plasmid constructs into a microbial host. Despite the availability of high efficiency bacterial hosts (achieving >10^9^ colony forming units per μg of DNA), the efficiency of transforming cloning or assembly reactions can be many orders of magnitude lower, primarily due to the relatively low efficiency of perfect plasmid assembly *in vitro*. For example, whilst Golden Gate is a reliable tool for the assembly of large constructs (often assembling >20 kb with over 20 parts), transformation of these assembly reactions typically yields 10^2^–10^3^ colonies (Coussement et al., 2017), with efficiency reducing further as the number of assembled parts increases (in contrast, the simple cloning of single gene parts in DE enables efficiencies of >10^6^ transformants). This limits the production of large combinatorial libraries with these methods, so effort is usually concentrated on the assembly of pre-selected construct designs in parallel. This dramatically increases resource requirements, is reliant on automation and reduces the screening scope. Therefore, improvements in assembly and cell transformation efficiency are required for the screening of large, more diverse synthetic pathway libraries. Alternatively, recent advancements in cell-free technologies could negate the need to conduct for transformations, and thereby increase the number of variants available for screening (Lu, 2017; Yehezkel et al., 2016).

For all of the approaches described here, the efficiency to the entire experimental process is greatly enhanced through integration with *in silico* design and learn strategies. At the design stage, integrating assembly designs with laboratory automation scripts greatly increases throughput and accuracy by reducing manual intervention steps (Carbonell et al., 2016). At the learn stage, machine learning algorithms provide a means to interrogate complex biological networks to unearth optimal sequences based on limited sampling of the design space. Machine learning has only recently been exploited for the DE of fluorescent proteins and enzymes (Fox, 2005; Mazurenko et al., 2020; Saito et al., 2018; Wu et al., 2016a; Wu et al., 2019b), and it has been tentatively explored in synthetic biology applications with small library sizes (Jervis et al., 2019; Opgenorth et al., 2019). If synthetic biology seeks to harness the potential of machine learning it will require the characterisation of large, diverse sequence libraries to provide enough data to generate meaningful predictions. To provide this, synthetic biology approaches must continue to improve assembly speed and accuracy, permitting a larger throughput of more diverse sequences. It is possible that advances in oligonucleotide synthesis technology could help in this regard. Protocols need to be streamlined for end-to-end automation, for example with use of one-pot assemblies or minimising complex liquid handling and DNA purification procedures. Statistical methods, like DoE, require smaller samples of sequence and system diversity and are a powerful means to optimise multifactorial problems to obtain improved function, albeit within more limited parameters than machine learning. It is envisaged that these computational approaches will provide a better understanding as to the key factors affecting the performance of biological molecules and systems. This knowledge could then be utilised to improve the predictability of biological engineering, a goal that can only be achieved through the accurate and efficient utilisation of genetic diversity.

## Declaration of Competing Interest

AC is the named inventor for a patent describing a mutagenesis method (GeneORator), which is mentioned in the manuscript.

## References

[bb0005] Acevedo-Rocha C.G., Reetz M.T., Nov Y. (2015). Economical analysis of saturation mutagenesis experiments. Sci. Rep..

[bb0010] Agresti J.J., Antipov E., Abate A.R., Ahn K., Rowat A.C., Baret J.-C., Marquez M., Klibanov A.M., Griffiths A.D., Weitz D.A. (2010). Ultrahigh-throughput screening in drop-based microfluidics for directed evolution. PNAS.

[bb0015] An Y., Ji J., Wu W., Lv A., Huang R., Wei Y. (2005). A rapid and efficient method for multiple-site mutagenesis with a modified overlap extension PCR. Appl. Microbiol. Biotechnol..

[bb0020] Arnold F.H. (1993). Protein engineering for unusual environments. Curr. Opin. Biotechnol..

[bb0025] Ashraf M., Frigotto L., Smith M.E., Patel S., Hughes M.D., Poole A.J., Hebaishi H.R.M., Ullman C.G., Hine A.V. (2013). ProxiMAX randomization: a new technology for non-degenerate saturation mutagenesis of contiguous codons. Biochem. Soc. Trans..

[bb0030] Autour A., Ryckelynck M. (2017). Ultrahigh-throughput improvement and discovery of enzymes using droplet-based microfluidic screening. Micromachines.

[bb0035] Bi Y., Qiao X., Hua Z., Zhang L., Liu X., Li L., Hua W., Xiao H., Zhou J., Wei Q., Zheng X. (2012). An asymmetric PCR-based, reliable and rapid single-tube native DNA engineering strategy. BMC Biotechnol..

[bb0040] Biggs B.W., De Paepe B., Santos C.N.S., De Mey M., Kumaran Ajikumar P. (2014). Multivariate modular metabolic engineering for pathway and strain optimization. Curr. Opin. Biotechnol. Cell Pathway Eng..

[bb0045] Biles B.D., Connolly B.A. (2004). Low-fidelity Pyrococcus furiosus DNA polymerase mutants useful in error-prone PCR. Nucleic Acids Res..

[bb0050] Breitling R., Takano E. (2015). Synthetic biology advances for pharmaceutical production. Current opinion in biotechnology. Chem. Biotechnol. Pharmaceut. Biotechnol..

[bb0055] Bryksin A.V., Matsumura I. (2010). Overlap extension PCR cloning: a simple and reliable way to create recombinant plasmids. Biotechniques.

[bb0060] Cadwell R.C., Joyce G.F. (1992). Randomization of genes by PCR mutagenesis. Genome Res..

[bb0065] Carbonell P., Currin A., Jervis A.J., Rattray N.J.W., Swainston N., Yan C., Takano E., Breitling R. (2016). Bioinformatics for the synthetic biology of natural products: integrating across the design–build–test cycle. Nat. Prod. Rep..

[bb0070] Carbonell P., Jervis A.J., Robinson C.J., Yan C., Dunstan M., Swainston N., Vinaixa M., Hollywood K.A., Currin A., Rattray N.J.W., Taylor S., Spiess R., Sung R., Williams A.R., Fellows D., Stanford N.J., Mulherin P., Feuvre R.L., Barran P., Goodacre R., Turner N.J., Goble C., Chen G.G., Kell D.B., Micklefield J., Breitling R., Takano E., Faulon J.-L., Scrutton N.S. (2018). An automated design-build-test-learn pipeline for enhanced microbial production of fine chemicals. Commun. Biol..

[bb0075] Caruthers M.H., Barone A.D., Beaucage S.L., Dodds D.R., Fisher E.F., McBride L.J., Matteucci M., Stabinsky Z., Tang J.-Y., Ray Wu L.G. (1987). [15] chemical synthesis of deoxyoligonucleotides by the phosphoramidite method. Methods in Enzymology, Recombinant DNA Part E.

[bb0080] Casini A., MacDonald J.T., Jonghe J.D., Christodoulou G., Freemont P.S., Baldwin G.S., Ellis T. (2013). One-pot DNA construction for synthetic biology: the modular overlap-directed assembly with linkers (MODAL) strategy. Nucleic Acids Res..

[bb0085] Casini A., Storch M., Baldwin G.S., Ellis T. (2015). Bricks and blueprints: methods and standards for DNA assembly. Nat. Rev. Mol. Cell Biol..

[bb0090] Cavaleiro A.M., Kim S.H., Seppälä S., Nielsen M.T., Nørholm M.H.H. (2015). Accurate DNA assembly and genome engineering with optimized uracil excision cloning. ACS Synth. Biol..

[bb0095] Chao R., Mishra S., Si T., Zhao H. (2017). Engineering biological systems using automated biofoundries. Metab. Eng..

[bb0100] Chen K.Q., Arnold F.H. (1991). Enzyme engineering for nonaqueous solvents: random mutagenesis to enhance activity of subtilisin E in polar organic media. Biotechnology (N.Y.).

[bb0105] Chen K., Arnold F.H. (1993). Tuning the activity of an enzyme for unusual environments: sequential random mutagenesis of subtilisin E for catalysis in dimethylformamide. Proc. Natl. Acad. Sci. U. S. A..

[bb0110] Cheng F., Zhu L., Schwaneberg U. (2015). Directed evolution 2.0: improving and deciphering enzyme properties. Chem. Commun..

[bb0115] Cheng F., Xu J.-M., Xiang C., Liu Z.-Q., Zhao L.-Q., Zheng Y.-G. (2017). Simple-MSSM: a simple and efficient method for simultaneous multi-site saturation mutagenesis. Biotechnol. Lett..

[bb0120] Cherry J.R., Fidantsef A.L. (2003). Directed evolution of industrial enzymes: an update. Curr. Opin. Biotechnol..

[bb0125] Chi H., Wang X., Shao Y., Qin Y., Deng Z., Wang L., Chen S. (2019). Engineering and modification of microbial chassis for systems and synthetic biology. Synthetic Syst. Biotechnol..

[bb0130] Cleary M.A., Kilian K., Wang Y., Bradshaw J., Cavet G., Ge W., Kulkarni A., Paddison P.J., Chang K., Sheth N., Leproust E., Coffey E.M., Burchard J., McCombie W.R., Linsley P., Hannon G.J. (2004). Production of complex nucleic acid libraries using highly parallel in situ oligonucleotide synthesis. Nat. Methods.

[bb0135] Copp Janine N., Hanson-Manful P., Ackerley David F., Patrick Wayne M., Gillam E.M.J., Copp J.N., Ackerley David (2014). Error-Prone PCR and effective generation of gene variant libraries for directed evolution. Directed Evolution Library Creation, Methods in Molecular Biology.

[bb0140] Coussement P., Bauwens D., Maertens J., De Mey M. (2017). Direct combinatorial pathway optimization. ACS Synth. Biol..

[bb0145] Cozens C., Pinheiro V.B. (2018). Darwin assembly: fast, efficient, multi-site bespoke mutagenesis. Nucleic Acids Res..

[bb0150] Currin A., Swainston N., Day P.J., Kell D.B. (2014). SpeedyGenes: an improved gene synthesis method for the efficient production of error-corrected, synthetic protein libraries for directed evolution. Protein Eng. Des. Sel..

[bb0155] Currin A., Swainston N., Day P.J., Kell D.B. (2015). Synthetic biology for the directed evolution of protein biocatalysts: navigating sequence space intelligently. Chem. Soc. Rev..

[bb0160] Currin A., Swainston N., Day P.J., Kell D.B. (2017). SpeedyGenes: exploiting an improved gene synthesis method for the efficient production of synthetic protein libraries for directed evolution. Methods Mol. Biol..

[bb0165] Currin A., Kwok J., Sadler J.C., Bell E.L., Swainston N., Ababi M., Day P., Turner N.J., Kell D.B. (2019). GeneORator: An effective strategy for navigating protein sequence space more efficiently through Boolean OR-type DNA libraries. ACS Synth. Biol..

[bb0170] Czar M.J., Anderson J.C., Bader J.S., Peccoud J. (2009). Gene synthesis demystified. Trends Biotechnol..

[bb0175] David F., Nielsen J., Siewers V. (2016). Flux control at the Malonyl-CoA node through hierarchical dynamic pathway regulation in Saccharomyces cerevisiae. ACS Synth. Biol..

[bb0180] de Kok S., Stanton L.H., Slaby T., Durot M., Holmes V.F., Patel K.G., Platt D., Shapland E.B., Serber Z., Dean J., Newman J.D., Chandran S.S. (2014). Rapid and reliable DNA assembly via ligase cycling reaction. ACS Synth. Biol..

[bb0185] Decoene T., Paepe B.D., Maertens J., Coussement P., Peters G., Maeseneire S.L.D., Mey M.D. (2018). Standardization in synthetic biology: an engineering discipline coming of age. Crit. Rev. Biotechnol..

[bb0190] Ding W., Weng H., Jin P., Du G., Chen J., Kang Z. (2017). Scarless assembly of unphosphorylated DNA fragments with a simplified DA℡ method. Bioengineered.

[bb0195] Dougherty M.J., Arnold F.H. (2009). Directed evolution: new parts and optimized function. Curr. Opin. Biotechnol..

[bb0200] Dueber J.E., Wu G.C., Malmirchegini G.R., Moon T.S., Petzold C.J., Ullal A.V., Prather K.L.J., Keasling J.D. (2009). Synthetic protein scaffolds provide modular control over metabolic flux. Nat. Biotechnol..

[bb0205] Eisenstein M. (2020). How to build a genome. Nature.

[bb0210] Ellis T., Adie T., Baldwin G.S. (2011). DNA assembly for synthetic biology: from parts to pathways and beyond. Integr. Biol..

[bb0215] Engler C., Kandzia R., Marillonnet S. (2008). A one pot, one step, precision cloning method with high throughput capability. PLoS One.

[bb0220] Engqvist M.K.M., Nielsen J. (2015). ANT: software for generating and evaluating degenerate codons for natural and expanded genetic codes. ACS Synth. Biol..

[bb0225] Foo J.L., Chang M.W. (2018). Synthetic yeast genome reveals its versatility. Nature.

[bb0230] Fox R. (2005). Directed molecular evolution by machine learning and the influence of nonlinear interactions. J. Theor. Biol..

[bb0235] Gao X., Yan P., Shen W., Li X., Zhou P., Li Y. (2013). Modular construction of plasmids by parallel assembly of linear vector components. Anal. Biochem..

[bb0240] Genee H.J., Bonde M.T., Bagger F.O., Jespersen J.B., Sommer M.O.A., Wernersson R., Olsen L.R. (2015). Software-supported USER cloning strategies for site-directed mutagenesis and DNA assembly. ACS Synth. Biol..

[bb0245] Gerosa L., Sauer U. (2011). Regulation and control of metabolic fluxes in microbes. Curr. Opin. Biotechnol. Nanobiotechnol. Syst. Biol..

[bb0250] Gerstmans H., Grimon D., Gutiérrez D., Lood C., Rodríguez A., van Noort V., Lammertyn J., Lavigne R., Briers Y. (2020). A VersaTile-driven platform for rapid hit-to-lead development of engineered lysins. Sci. Adv..

[bb0255] Geu-Flores F., Nour-Eldin H.H., Nielsen M.T., Halkier B.A. (2007). USER fusion: a rapid and efficient method for simultaneous fusion and cloning of multiple PCR products. Nucleic Acids Res..

[bb0260] Gibson D.G. (2009). Synthesis of DNA fragments in yeast by one-step assembly of overlapping oligonucleotides. Nucleic Acids Res..

[bb0265] Gibson D.G., Voigt Christopher (2011). Chapter fifteen - enzymatic assembly of overlapping DNA fragments. Methods in Enzymology.

[bb0270] Gibson D.G., Benders G.A., Andrews-Pfannkoch C., Denisova E.A., Baden-Tillson H., Zaveri J., Stockwell T.B., Brownley A., Thomas D.W., Algire M.A., Merryman C., Young L., Noskov V.N., Glass J.I., Venter J.C., Hutchison C.A., Smith H.O. (2008). Complete chemical synthesis, assembly, and cloning of a mycoplasma genitalium genome. Science.

[bb0275] Gibson D.G., Young L., Chuang R.-Y., Venter J.C., Hutchison C.A., Smith H.O. (2009). Enzymatic assembly of DNA molecules up to several hundred kilobases. Nat. Methods.

[bb0280] Gibson D.G., Smith H.O., Iii C.A.H., Venter J.C., Merryman C. (2010). Chemical synthesis of the mouse mitochondrial genome. Nat. Methods.

[bb0285] Goldenzweig A., Goldsmith M., Hill S.E., Gertman O., Laurino P., Ashani Y., Dym O., Unger T., Albeck S., Prilusky J., Lieberman R.L., Aharoni A., Silman I., Sussman J.L., Tawfik D.S., Fleishman S.J. (2016). Automated structure- and sequence-based Design of Proteins for high bacterial expression and stability. Mol. Cell.

[bb0290] Halleran A.D., Swaminathan A., Murray R.M. (2018). Single Day construction of multigene circuits with 3G assembly. ACS Synth. Biol..

[bb0295] Halweg-Edwards A.L., Pines G., Winkler J.D., Pines A., Gill R.T. (2016). A web Interface for codon compression. ACS Synth. Biol..

[bb0300] HamediRad M., Weisberg S., Chao R., Lian J., Zhao H. (2019). Highly efficient single-pot Scarless Golden Gate assembly. ACS Synth. Biol..

[bb0305] Hayashi Y., Aita T., Toyota H., Husimi Y., Urabe I., Yomo T. (2006). Experimental rugged fitness landscape in protein sequence space. PLoS One.

[bb0310] Heckman K.L., Pease L.R. (2007). Gene splicing and mutagenesis by PCR-driven overlap extension. Nat. Protoc..

[bb0315] Hillson N., Caddick M., Cai Y., Carrasco J.A., Chang M.W., Curach N.C., Bell D.J., Le Feuvre R., Friedman D.C., Fu X., Gold N.D., Herrgård M.J., Holowko M.B., Johnson J.R., Johnson R.A., Keasling J.D., Kitney R.I., Kondo A., Liu C., Martin V.J.J., Menolascina F., Ogino C., Patron N.J., Pavan M., Poh C.L., Pretorius I.S., Rosser S.J., Scrutton N.S., Storch M., Tekotte H., Travnik E., Vickers C.E., Yew W.S., Yuan Y., Zhao H., Freemont P.S. (2019). Building a global alliance of biofoundries. Nat. Commun..

[bb0320] Hoebenreich S., Zilly F.E., Acevedo-Rocha C.G., Zilly M., Reetz M.T. (2014). Speeding up directed evolution: combining the advantages of solid-phase combinatorial gene synthesis with statistically guided reduction of screening effort. ACS Synth. Biol..

[bb0325] Hughes R.A., Miklos A.E., Ellington A.D., Voigt Christopher (2011). Chapter twelve - gene synthesis: methods and applications. Methods in Enzymology.

[bb0330] Hussain H., Chong N.F.-M. (2016). Combined overlap extension PCR method for improved site directed mutagenesis. Biomed. Res. Int..

[bb0335] Hutchison C.A., Phillips S., Edgell M.H., Gillam S., Jahnke P., Smith M. (1978). Mutagenesis at a specific position in a DNA sequence. J. Biol. Chem..

[bb0340] Itakura K., Rossi J.J., Wallace R.B. (1984). Synthesis and use of synthetic oligonucleotides. Annu. Rev. Biochem..

[bb0345] Iverson S.V., Haddock T.L., Beal J., Densmore D.M. (2016). CIDAR MoClo: improved MoClo assembly standard and new E. coli part library enable rapid combinatorial Design for Synthetic and Traditional Biology. ACS Synth. Biol..

[bb0350] Jacobs T.M., Yumerefendi H., Kuhlman B., Leaver-Fay A. (2015). SwiftLib: rapid degenerate-codon-library optimization through dynamic programming. Nucleic Acids Res..

[bb0355] Jervis A.J., Carbonell P., Vinaixa M., Dunstan M.S., Hollywood K.A., Robinson C.J., Rattray N.J.W., Yan C., Swainston N., Currin A., Sung R., Toogood H., Taylor S., Faulon J.-L., Breitling R., Takano E., Scrutton N.S. (2019). Machine learning of designed translational control allows predictive pathway optimization in Escherichia coli. ACS Synth. Biol..

[bb0360] Jeschek M., Gerngross D., Panke S. (2016). Rationally reduced libraries for combinatorial pathway optimization minimizing experimental effort. Nat. Commun..

[bb0365] Jeschek M., Gerngross D., Panke S. (2017). Combinatorial pathway optimization for streamlined metabolic engineering. Curr. Opin. Biotechnol. Tissue Cell Pathway Eng..

[bb0370] Jia B., Wu Y., Li B.-Z., Mitchell L.A., Liu H., Pan S., Wang J., Zhang H.-R., Jia N., Li B., Shen M., Xie Z.-X., Liu D., Cao Y.-X., Li X., Zhou X., Qi H., Boeke J.D., Yuan Y.-J. (2018). Precise control of SCRaMbLE in synthetic haploid and diploid yeast. Nat. Commun..

[bb0375] Jin P., Ding W., Du G., Chen J., Kang Z. (2016). DATEL: a Scarless and sequence-independent DNA assembly method using Thermostable exonucleases and ligase. ACS Synth. Biol..

[bb0380] Jin P., Kang Z., Zhang J., Zhang L., Du G., Chen J. (2016). Combinatorial evolution of enzymes and synthetic pathways using one-step PCR. ACS Synth. Biol..

[bb0385] Khalil A.S., Collins J.J. (2010). Synthetic biology: applications come of age. Nat. Rev. Genet..

[bb0390] Khersonsky O., Lipsh R., Avizemer Z., Ashani Y., Goldsmith M., Leader H., Dym O., Rogotner S., Trudeau D.L., Prilusky J., Amengual-Rigo P., Guallar V., Tawfik D.S., Fleishman S.J. (2018). Automated design of efficient and functionally diverse enzyme repertoires. Mol. Cell.

[bb0395] Kikuchi M., Ohnishi K., Harayama S. (1999). Novel family shuffling methods for the in vitro evolution of enzymes. Gene.

[bb0400] Kille S., Acevedo-Rocha C.G., Parra L.P., Zhang Z.-G., Opperman D.J., Reetz M.T., Acevedo J.P. (2013). Reducing codon redundancy and screening effort of combinatorial protein libraries created by saturation mutagenesis. ACS Synth. Biol..

[bb0405] Kondrashov D.A., Kondrashov F.A. (2015). Topological features of rugged fitness landscapes in sequence space. Trends Genet..

[bb0410] LeProust E., Zhang H., Yu P., Zhou X., Gao X. (2001). Characterization of oligodeoxyribonucleotide synthesis on glass plates. Nucleic Acids Res..

[bb0415] LeProust E.M., Peck B.J., Spirin K., McCuen H.B., Moore B., Namsaraev E., Caruthers M.H. (2010). Synthesis of high-quality libraries of long (150mer) oligonucleotides by a novel depurination controlled process. Nucleic Acids Res..

[bb0420] Li A., Acevedo-Rocha C.G., Sun Z., Cox T., Xu J.L., Reetz M.T. (2018). Beating Bias in the directed evolution of proteins: combining high-Fidelity on-Chip solid-phase gene synthesis with efficient gene assembly for combinatorial library construction. ChemBioChem.

[bb0425] Li H., Huang Y., Wei Z., Wang W., Yang Z., Liang Z., Li Z. (2019). An oligonucleotide synthesizer based on a microreactor chip and an inkjet printer. Sci. Rep..

[bb0430] Lu Y. (2017). Cell-free synthetic biology: engineering in an open world. Synthetic and Systems Biotechnology, A tribute to Arny Demain, for his lifelong pioneering contributions to biochemical engineering.

[bb0435] Ma S., Saaem I., Tian J. (2012). Error correction in gene synthesis technology. Trends Biotechnol..

[bb0440] Ma L., Li Y., Chen X., Ding M., Wu Y., Yuan Y.-J. (2019). SCRaMbLE generates evolved yeasts with increased alkali tolerance. Microb. Cell Factories.

[bb0445] Mao Y., Lin J., Zhou A., Ji K., Downey J.S., Chen R., Han A. (2011). Quikgene: a gene synthesis method integrated with ligation-free cloning. Anal. Biochem..

[bb0450] Mazurenko S., Prokop Z., Damborsky J. (2020). Machine learning in enzyme engineering. ACS Catal..

[bb0455] McCullum E.O., Williams B.A.R., Zhang J., Chaput J.C., Braman J. (2010). Random mutagenesis by error-prone PCR. In Vitro Mutagenesis Protocols, Methods in Molecular Biology.

[bb0460] Miton C.M., Tokuriki N. (2016). How mutational epistasis impairs predictability in protein evolution and design. Protein Sci..

[bb0465] Moore S.J., Lai H.-E., Kelwick R.J.R., Chee S.M., Bell D.J., Polizzi K.M., Freemont P.S. (2016). EcoFlex: a multifunctional MoClo kit for E. coli synthetic biology. ACS Synth. Biol..

[bb0470] Naseri G., Koffas M.A.G. (2020). Application of combinatorial optimization strategies in synthetic biology. Nat. Commun..

[bb0475] Nour-Eldin H.H., Hansen B.G., Nørholm M.H.H., Jensen J.K., Halkier B.A. (2006). Advancing uracil-excision based cloning towards an ideal technique for cloning PCR fragments. Nucleic Acids Res..

[bb0480] Nov Y. (2012). When second best is good enough: another probabilistic look at saturation mutagenesis. Appl. Environ. Microbiol..

[bb0485] Opgenorth P., Costello Z., Okada T., Goyal G., Chen Y., Gin J., Benites V., de Raad M., Northen T.R., Deng K., Deutsch S., Baidoo E.E.K., Petzold C.J., Hillson N.J., Garcia Martin H., Beller H.R. (2019). Lessons from two design–build–test–learn cycles of Dodecanol production in Escherichia coli aided by machine learning. ACS Synth. Biol..

[bb0490] Pines G., Pines A., Garst A.D., Zeitoun R.I., Lynch S.A., Gill R.T. (2015). Codon compression algorithms for saturation mutagenesis. ACS Synth. Biol..

[bb0495] Popova B., Schubert S., Bulla I., Buchwald D., Kramer W. (2015). A robust and Versatile method of combinatorial chemical synthesis of gene libraries via hierarchical assembly of partially randomized modules. PLoS One.

[bb0500] Potapov V., Ong J.L., Kucera R.B., Langhorst B.W., Bilotti K., Pryor J.M., Cantor E.J., Canton B., Knight T.F., Evans T.C., Lohman G.J.S. (2018). Comprehensive profiling of four base overhang ligation Fidelity by T4 DNA ligase and application to DNA assembly. ACS Synth. Biol..

[bb0505] Povolotskaya I.S., Kondrashov F.A. (2010). Sequence space and the ongoing expansion of the protein universe. Nature.

[bb0510] Pretorius I.S., Boeke J.D. (2018). Yeast 2.0-connecting the dots in the construction of the world’s first functional synthetic eukaryotic genome. FEMS Yeast Res..

[bb0515] Qu G., Li A., Acevedo-Rocha C.G., Sun Z., Reetz M.T. (2020). The crucial role of methodology development in directed evolution of selective enzymes. Angew. Chem. Int. Ed..

[bb0520] Quaglia D., Ebert M.C.C.J.C., Mugford P.F., Pelletier J.N. (2017). Enzyme engineering: a synthetic biology approach for more effective library generation and automated high-throughput screening. PLoS One.

[bb0525] Reetz M.T., Wang L.-W., Bocola M. (2006). Directed evolution of Enantioselective enzymes: iterative cycles of CASTing for probing protein-sequence space. Angew. Chem. Int. Ed..

[bb0530] Reetz M.T., Kahakeaw D., Lohmer R. (2008). Addressing the numbers problem in directed evolution. ChemBioChem.

[bb0535] Robinson C.J., Dunstan M.S., Swainston N., Titchmarsh J., Takano E., Scrutton N.S., Jervis A.J., Scrutton N. (2018). Multifragment DNA assembly of biochemical pathways via automated ligase cycling reaction. Methods in Enzymology, Enzymes in Synthetic Biology.

[bb0540] Robinson C.J., Carbonell P., Jervis A.J., Yan C., Hollywood K.A., Dunstan M.S., Currin A., Swainston N., Spiess R., Taylor S., Mulherin P., Parker S., Rowe W., Matthews N.E., Malone K.J., Le Feuvre R., Shapira P., Barran P., Turner N.J., Micklefield J., Breitling R., Takano E., Scrutton N.S. (2020). Rapid prototyping of microbial production strains for the biomanufacture of potential materials monomers. Metab. Eng..

[bb0545] Romero P.A., Arnold F.H. (2009). Exploring protein fitness landscapes by directed evolution. Nat. Rev. Mol. Cell Biol..

[bb0550] Romero P.A., Krause A., Arnold F.H. (2013). Navigating the protein fitness landscape with Gaussian processes. PNAS.

[bb0555] Sadler J.C., Green L., Swainston N., Kell D.B., Currin A. (2018). Fast and flexible synthesis of combinatorial libraries for directed evolution. Methods in Enzymology.

[bb0560] Sailer Z.R., Harms M.J. (2018). Uninterpretable Interact. Epistasis Uncertainty.

[bb0565] Saito Y., Oikawa M., Nakazawa H., Niide T., Kameda T., Tsuda K., Umetsu M. (2018). Machine-learning-guided mutagenesis for directed evolution of fluorescent proteins. ACS Synth. Biol..

[bb0570] Santos-Moreno J., Schaerli Y. (2019). A framework for the modular and combinatorial assembly of synthetic gene circuits. ACS Synth. Biol..

[bb0575] Sayous V., Lubrano P., Li Y., Acevedo-Rocha C.G. (2020). Unbiased libraries in protein directed evolution. Biochimica et Biophysica Acta (BBA) - Proteins and Proteomics.

[bb0580] Schindler D., Dai J., Cai Y. (2018). Synthetic genomics: a new venture to dissect genome fundamentals and engineer new functions. Curr. Opin. Chem. Biol. Synthetic Biol. Synthetic Biomol..

[bb0585] Schmidt T.L., Beliveau B.J., Uca Y.O., Theilmann M., Da Cruz F., Wu C.-T., Shih W.M. (2015). Scalable amplification of strand subsets from chip-synthesized oligonucleotide libraries. Nat. Commun..

[bb0590] Shao Z., Zhao H., Polizzi K.M., Kontoravdi C. (2013). Construction and engineering of large biochemical pathways via DNA assembler. Synthetic Biology, Methods in Molecular Biology.

[bb0595] Shao Z., Zhao Hua, Zhao Huimin (2009). DNA assembler, an in vivo genetic method for rapid construction of biochemical pathways. Nucleic Acids Res..

[bb0600] Simon A.J., d’Oelsnitz S., Ellington A.D. (2019). Synthetic evolution. Nat. Biotechnol..

[bb0605] Smanski M.J., Bhatia S., Zhao D., Park Y., Woodruff L.B.A., Giannoukos G., Ciulla D., Busby M., Calderon J., Nicol R., Gordon D.B., Densmore D., Voigt C.A. (2014). Functional optimization of gene clusters by combinatorial design and assembly. Nat. Biotechnol..

[bb0610] Smanski M.J., Zhou H., Claesen J., Shen B., Fischbach M.A., Voigt C.A. (2016). Synthetic biology to access and expand nature’s chemical diversity. Nat. Rev. Microbiol..

[bb0615] Solomon K.V., Sanders T.M., Prather K.L.J. (2012). A dynamic metabolite valve for the control of central carbon metabolism. Metab. Eng..

[bb0620] Stemmer W.P. (1994). DNA shuffling by random fragmentation and reassembly: in vitro recombination for molecular evolution. PNAS.

[bb0625] Stemmer W.P.C. (1994). Rapid evolution of a protein in vitro by DNA shuffling. Nature.

[bb0630] Storch M., Casini A., Mackrow B., Fleming T., Trewhitt H., Ellis T., Baldwin G.S. (2015). BASIC: a new biopart assembly standard for idempotent cloning provides accurate, Single-Tier DNA Assembly for Synthetic Biology. ACS Synth. Biol..

[bb0635] Storch M., Casini A., Mackrow B., Ellis T., Baldwin G.S., Hughes R.A. (2017). BASIC: A simple and accurate modular DNA assembly method. Synthetic DNA: Methods and Protocols, Methods in Molecular Biology.

[bb0640] Storz J.F. (2018). Compensatory mutations and epistasis for protein function. Current Opinion in Structural Biology, Carbohydrates Sequences and Topology.

[bb0645] Sumbalova L., Stourac J., Martinek T., Bednar D., Damborsky J. (2018). HotSpot wizard 3.0: web server for automated design of mutations and smart libraries based on sequence input information. Nucleic Acids Res..

[bb0650] Swainston N., Currin A., Green L., Breitling R., Day P.J., Kell D.B. (2017). CodonGenie: optimised ambiguous codon design tools. PeerJ Comput. Sci..

[bb0655] Tang L., Gao H., Zhu X., Wang X., Zhou M., Jiang R. (2012). Construction of “small-intelligent” focused mutagenesis libraries using well-designed combinatorial degenerate primers. BioTechniques.

[bb0660] Taylor G.M., Mordaka P.M., Heap J.T. (2019). Start-stop assembly: a functionally scarless DNA assembly system optimized for metabolic engineering. Nucleic Acids Res..

[bb0665] Tian J., Gong H., Sheng N., Zhou X., Gulari E., Gao X., Church G. (2004). Accurate multiplex gene synthesis from programmable DNA microchips. Nature.

[bb0670] Torella J.P., Boehm C.R., Lienert F., Chen J.-H., Way J.C., Silver P.A. (2014). Rapid construction of insulated genetic circuits via synthetic sequence-guided isothermal assembly. Nucleic Acids Res..

[bb0675] Tracewell C.A., Arnold F.H. (2009). Directed enzyme evolution: climbing fitness peaks one amino acid at a time. Curr. Opin. Chem. Biol..

[bb0680] Trubitsyna M., Michlewski G., Cai Y., Elfick A., French C.E. (2014). PaperClip: rapid multi-part DNA assembly from existing libraries. Nucleic Acids Res..

[bb0685] Trubitsyna M., Liu C.-K., Salinas A., Elfick A., French C.E., Hughes R.A. (2017). PaperClip: A simple method for flexible multi-part DNA assembly. Synthetic DNA: Methods and Protocols, Methods in Molecular Biology.

[bb0690] Van den Brulle J., Fischer M., Langmann T., Horn G., Waldmann T., Arnold S., Fuhrmann M., Schatz O., O’Connell T., O’Connell D., Auckenthaler A., Schwer H. (2008). A novel solid phase technology for high-throughput gene synthesis. BioTechniques.

[bb0695] van Dolleweerd C.J., Kessans S.A., Van de Bittner K.C., Bustamante L.Y., Bundela R., Scott B., Nicholson M.J., Parker E.J. (2018). MIDAS: a modular DNA assembly system for synthetic biology. ACS Synth. Biol..

[bb0700] Vecchione S., Fritz G. (2019). CRIMoClo plasmids for modular assembly and orthogonal chromosomal integration of synthetic circuits in Escherichia coli. J. Biol. Eng..

[bb0705] Wäneskog M., Bjerling P. (2014). Multi-fragment site-directed mutagenic overlap extension polymerase chain reaction as a competitive alternative to the enzymatic assembly method. Anal. Biochem..

[bb0710] Wang X., Lin H., Zheng Y., Feng J., Yang Z., Tang L. (2015). MDC-analyzer-facilitated combinatorial strategy for improving the activity and stability of halohydrin dehalogenase from agrobacterium radiobacter AD1. J. Biotechnol..

[bb0715] Weber E., Engler C., Gruetzner R., Werner S., Marillonnet S. (2011). A modular cloning system for standardized assembly of multigene constructs. PLoS One.

[bb0720] Wei H., Hu J., Wang L., Xu F., Wang S. (2012). Rapid gene splicing and multi-sited mutagenesis by one-step overlap extension polymerase chain reaction. Anal. Biochem..

[bb0725] Weinstein J., Khersonsky O., Fleishman S.J. (2020). Practically useful protein-design methods combining phylogenetic and atomistic calculations. Curr. Opin. Struct. Biol..

[bb0730] Werner S., Engler C., Weber E., Gruetzner R., Marillonnet S. (2012). Fast track assembly of multigene constructs using Golden Gate cloning and the MoClo system. Bioengineered.

[bb0735] Wiedmann M., Wilson W.J., Czajka J., Luo J., Barany F., Batt C.A. (1994). Ligase chain reaction (LCR)-overview and applications. Genome Res..

[bb0740] Williams E.M., Copp J.N., Ackerley D.F., Gillam E.M.J., Copp J.N., Ackerley D. (2014). Site-saturation mutagenesis by overlap extension PCR. Directed Evolution Library Creation: Methods and Protocols, Methods in Molecular Biology.

[bb0745] Wong T.S., Roccatano D., Zacharias M., Schwaneberg U. (2006). A statistical analysis of random mutagenesis methods used for directed protein evolution. J. Mol. Biol..

[bb0750] Wong T.S., Wong T.S., Roccatano D., Schwaneberg U. (2007). Challenges of the genetic code for exploring sequence space in directed protein evolution. Biocatal Biotransformation.

[bb0755] Wu J., Du G., Chen J., Zhou J. (2015). Enhancing flavonoid production by systematically tuning the central metabolic pathways based on a CRISPR interference system in *Escherichia coli*. Sci. Rep..

[bb0760] Wu S.G., Shimizu K., Tang J.K.-H., Tang Y.J. (2016). Facilitate collaborations among synthetic biology, metabolic engineering and machine learning. ChemBioEng Rev..

[bb0765] Wu G., Yan Q., Jones J.A., Tang Y.J., Fong S.S., Koffas M.A.G. (2016). Metabolic burden: cornerstones in synthetic biology and metabolic engineering applications. Trends Biotechnol..

[bb0770] Wu Y., Zhu R.-Y., Mitchell L.A., Ma L., Liu R., Zhao M., Jia B., Xu H., Li Y.-X., Yang Z.-M., Ma Y., Li X., Liu H., Liu D., Xiao W.-H., Zhou X., Li B.-Z., Yuan Y.-J., Boeke J.D. (2018). In vitro DNA SCRaMbLE. Nat. Commun..

[bb0775] Wu M.-R., Jusiak B., Lu T.K. (2019). Engineering advanced cancer therapies with synthetic biology. Nat. Rev. Cancer.

[bb0780] Wu Z., Kan S.B.J., Lewis R.D., Wittmann B.J., Arnold F.H. (2019). Machine learning-assisted directed protein evolution with combinatorial libraries. PNAS.

[bb0785] Xia Y., Chu W., Qi Q., Xun L. (2015). New insights into the QuikChangeTM process guide the use of Phusion DNA polymerase for site-directed mutagenesis. Nucleic Acids Res..

[bb0790] Xia Y., Li K., Li J., Wang T., Gu L., Xun L. (2019). T5 exonuclease-dependent assembly offers a low-cost method for efficient cloning and site-directed mutagenesis. Nucleic Acids Res..

[bb0795] Xiao Y.-H., Pei Y., Park D.J. (2011). Asymmetric overlap extension PCR method for site-directed mutagenesis. PCR Protocols, Methods in Molecular Biology.

[bb0800] Xu P., Rizzoni E.A., Sul S.-Y., Stephanopoulos G. (2017). Improving metabolic pathway efficiency by statistical model-based multivariate regulatory metabolic engineering. ACS Synth. Biol..

[bb0805] Yang J., Ruff A.J., Arlt M., Schwaneberg U. (2017). Casting epPCR (cepPCR): a simple random mutagenesis method to generate high quality mutant libraries. Biotechnol. Bioeng..

[bb0810] Yehezkel T.B., Rival A., Raz O., Cohen R., Marx Z., Camara M., Dubern J.-F., Koch B., Heeb S., Krasnogor N., Delattre C., Shapiro E. (2016). Synthesis and cell-free cloning of DNA libraries using programmable microfluidics. Nucleic Acids Res..

[bb0815] Yuan Y., Andersen E., Zhao H. (2016). Flexible and Versatile strategy for the construction of large biochemical pathways. ACS Synth. Biol..

[bb0820] Zhao H., Arnold F.H. (1997). Optimization of DNA shuffling for high Fidelity recombination. Nucleic Acids Res..

[bb0825] Zhou H., Vonk B., Roubos J.A., Bovenberg R.A.L., Voigt C.A. (2015). Algorithmic co-optimization of genetic constructs and growth conditions: application to 6-ACA, a potential nylon-6 precursor. Nucleic Acids Res..

